# ‘You’re never pregnant in the same way again’: prior early pregnancy loss influences need for health care and support in subsequent pregnancy

**DOI:** 10.1093/hropen/hoad032

**Published:** 2023-08-01

**Authors:** E Koert, T S Hartwig, G M Hviid Malling, L Schmidt, H S Nielsen

**Affiliations:** Department of Public Health, Section of Social Medicine, University of Copenhagen, Copenhagen K, Denmark; Department of Obstetrics and Gynecology, Amager Hvidovre Hospital, Copenhagen University Hospital, Hvidovre, Denmark; Department of Obstetrics and Gynecology, Amager Hvidovre Hospital, Copenhagen University Hospital, Hvidovre, Denmark; Department of Public Health, Section of Social Medicine, University of Copenhagen, Copenhagen K, Denmark; Department of Public Health, Section of Social Medicine, University of Copenhagen, Copenhagen K, Denmark; Department of Obstetrics and Gynecology, Amager Hvidovre Hospital, Copenhagen University Hospital, Recurrent Pregnancy Loss Unit, Hvidovre, Denmark

**Keywords:** early pregnancy loss, pregnancy, pregnancy care, longitudinal study, psychosocial factors

## Abstract

**STUDY QUESTION:**

What are couples’ needs for health care and support in a subsequent pregnancy after prior early pregnancy loss (PL) and how do needs change across the pregnancy?

**SUMMARY ANSWER:**

Couples described unmet needs for pregnancy care in the first 20 weeks of pregnancy and were more satisfied with the care provided during the remainder of the pregnancy.

**WHAT IS KNOWN ALREADY:**

Despite early PL being common (∼25% of pregnancies), there is a paucity of research to guide practice to optimize treatment and support future pregnancies. There has been low priority for the issue in research and a pervasive acceptance that couples should ‘just try again’ after experiencing PL. Women with prior PL report increased anxiety during the first trimester of pregnancy compared to those without previous PL. No longitudinal studies explore what couples’ needs are throughout the pregnancy and how these needs shift across time.

**STUDY DESIGN, SIZE, DURATION:**

This was a qualitative longitudinal dyadic (joint) interview study. In total, 15 couples who were pregnant after a prior PL were interviewed four times over their pregnancy. Couples were recruited from the Copenhagen Pregnancy Loss Cohort Research Programme. Interviews were held in person at the hospital or university, or online. Interviews ranged from 20 to 91 min (mean = 54 min).

**PARTICIPANTS/MATERIALS, SETTING, METHODS:**

Inclusion criteria included couples with one to two prior early PL(s) who self-reported a new pregnancy and were willing to be interviewed together and in English. Couples were interviewed four times: after a positive pregnancy test and once in each trimester. Interviews were transcribed and data were analysed using thematic analysis to compare and contrast needs of the couples at each of the four time periods in the pregnancy and across the entire pregnancy. One same-sex couple and 14 heterosexual couples participated.

**MAIN RESULTS AND THE ROLE OF CHANCE:**

Couples’ needs were categorized into two main longitudinal themes across the pregnancy, divided by the 20-week scan. Within each longitudinal theme, there were two themes to represent each time period. In the longitudinal theme ‘*The first 20 weeks: a ‘scary’ gap in care*’ there were two themes: *Positive pregnancy test: ‘Tell them it’s not the same pregnancy’* and *First trimester: ‘We craved that someone was taking care of us’.* The standard pregnancy care offered in the public healthcare system in Denmark includes a scan at 12 and 20 weeks. While all couples wished for additional access to scans and monitoring of the foetus in early pregnancy to provide reassurance and detect problems early, they described considerable variation in the referrals and care they were offered. Both partners expressed a high degree of worry and anxiety about the pregnancy, with pregnant women in particular describing ‘surviv[ing] from scan to scan’ in the early weeks. Couples took scans wherever offered or paid for comfort scans, but this resulted in fragmented care. Instead, they wished for continuity in care, and acknowledgement and sensitivity that a pregnancy after PL is not the same as a first pregnancy. In the longitudinal theme ‘*The second 20 weeks: Safety in the care system*’ there were two themes: *Second trimester: ‘I think we are in good hands’* and *Third trimester: ‘It’s more of a ‘nice to know’ everything is OK than a ‘need to know’. C*ouples reported their distress was lower and overall needs for care were met during this time. They expressed general satisfaction with regular or extended antenatal support although, as in the first 20 weeks, additional acknowledgement and sensitivity regarding their history of PL was desired. Couples said they felt more secure given that they had access to a 24-hour telephone support by midwife/nurse if they had any concerns or questions.

**LIMITATIONS, REASONS FOR CAUTION:**

Participants were self-selected from an ongoing cohort study of patients presenting at hospital with PL. Single women were not included in the study. This study was limited to data collection in Denmark; however, other countries with public healthcare systems may have similar offerings with regard to their provision of antenatal care, care provided in recurrent pregnancy loss (RPL) clinics and the availability of private scans.

**WIDER IMPLICATIONS OF THE FINDINGS:**

The findings underscore that an early PL creates an increased need for monitoring and care in a subsequent pregnancy. This study highlights a gap in pregnancy care for those with a history of PL given that their need for monitoring and support is high in the early weeks of a new pregnancy before they have access to antenatal care, and before they have had multiple PLs and can be referred to the RPL unit.

**STUDY FUNDING/COMPETING INTEREST(S):**

This project has received funding from the European Union’s Horizon 2020 research and innovation programme under the Marie Skłodowska-Curie grant agreement No 101028172 for E.K. The Copenhagen Pregnancy Loss Cohort is funded by a grant from the BioInnovation Institute Foundation. H.S.N. has received scientific grants from Freya Biosciences, Ferring Pharmaceuticals, BioInnovation Institute, Ministry of Education, Novo Nordisk Foundation, Augustinus Fonden, Oda og Hans Svenningsens Fond, Demant Fonden, Ole Kirks Fond, and Independent Research Fund Denmark. H.S.N. received personal payment or honoraria for lectures and presentations from Ferring Pharmaceuticals, Merck, Astra Zeneca, Cook Medical, Gedeon Richter, and Ibsa Nordic. All other authors declare no competing interests.

WHAT DOES THIS MEAN FOR PATIENTS? Pregnancy loss is common; thus, there has been an overall assumption that couples should ‘just try again’ after a pregnancy loss, which minimizes the psychological and physical impact of this event. However, this repeated interview study, conducted with couples that were pregnant after one or more prior pregnancy loss(es), demonstrates that an early pregnancy loss is not an isolated event and that it can affect both partners in a next pregnancy. The study findings show that couples believe there is a ‘scary’ gap in the pregnancy care provided in the early weeks of a subsequent pregnancy when they do not have access to antenatal care (generally available in the second half of pregnancy) and they are not eligible to attend the recurrent pregnancy loss unit. During this time, both partners’ anxiety and distress is high, given their concern that they may experience another loss. These findings underscore that care after pregnancy loss should extend into the next early pregnancy. This study also shows that after the 20-week mark, couples feel safety in the antenatal care that is provided and more secure about the pregnancy as the weeks progress.

## Introduction

Pregnancy loss (PL) is common, ending ∼25% of all recognized pregnancies (Nybo Andersen *et al.*, 2000; Macklon *et al.*, 2002; Quenby *et al.*, 2021). A PL is defined as the spontaneous loss of a pregnancy before the foetus is viable (Bender Atik *et al*., 2023). The upper gestational age for PL differs across countries. In Denmark, it is defined as spontaneous loss before 22 weeks gestation whilst ‘early’ PL is before the end of the first trimester (i.e. 12 weeks) (Hartwig *et al.*, 2023). There has been a paucity of research to guide practice to optimize treatment and support future pregnancies after sporadic PL. There has been low priority in research and pervasive acceptance that couples should ‘just try again’ after experiencing one or two PL(s) (The Lancet, 2021a). But as outlined in the 2021 *The Lancet* series, *Miscarriage Matters*, research on the short- and long-term health consequences of PL ‘challenge[s] the belief that miscarriage is a single event without wider repercussions’ (The Lancet, 2021a). This series concludes with the recommendation that miscarriage care needs a worldwide reform that reflects this significant physical and psychological event.

There is now strong evidence of the short- and long-term negative mental health and psychosocial implications of PL for women and their partners (Brier, 2008; Kolte *et al.*, 2015; Meaney *et al.*, 2017; Koert *et al.*, 2019; Obst *et al.*, 2020; Williams *et al.*, 2020; Quenby *et al.*, 2021). There is some evidence that women experience higher anxiety and pregnancy-related distress in a new pregnancy after prior PL in comparison to women without PL (Bergner *et al.*, 2008; McCarthy *et al.*, 2015; Hunter *et al.*, 2017; Shapiro *et al.*, 2017; Lazarides *et al.*, 2021), in particular in the first trimester. These findings are concerning given that studies have shown that pregnancy-specific anxiety may be associated with an increased risk of negative pregnancy outcomes, such as preterm birth and low birthweight, with implications for infant health and well-being (Lobel *et al.*, 2008; Dunkel Schetter *et al*., 2022). Few longitudinal studies measure how anxiety and distress change across a pregnancy. Even fewer studies include and measure partners’ distress during a pregnancy (Hunter *et al.*, 2017). This makes it difficult to understand when women and partners are in most need of targeted care after sporadic PL. This knowledge is needed in order to promote overall well-being and best outcomes for couples and their future child.

Research shows that women and their partners are unhappy with the existing treatment and care that they receive after PL and wish for individualized care including information, follow-up, and support (Musters *et al.*, 2011; Meaney *et al.*, 2017; van den Berg *et al.*, 2018). A scoping review found that women and men were dissatisfied with the emotional support they received in hospitals after PL and found that some factors, such as lack of information and support from health professionals, exacerbated their emotional distress (Galeotti *et al.*, 2022). Even less is known about their needs in a new pregnancy and their perceptions of the care they receive during this phase. A cross-sectional qualitative study conducted by our group on couples’ perspectives on their need for treatment, support, and follow-up after recurrent pregnancy loss (RPL) indicated that they wished for ongoing monitoring in a new pregnancy after one or two PL(s), before they could access the monitoring offered in the RPL Unit (Koert *et al*., 2019).

One of *The Lancet* series, *Miscarriage Matters*, recommendations for worldwide reform of PL care is that early pregnancy monitoring should be standard care after sporadic (1–2) PLs (Coomarasamy *et al.*, 2021). However, included in a reform of care should be an examination of patients’ perspectives on their needs for care, and whether and how these needs persist across the pregnancy.

An exploratory qualitative study is appropriate when little is known about an experience (Patton, 2015). A longitudinal design allows for the examination of changes in needs over time and dyadic interviews are appropriate to examine a shared experience and needs within couples (Saldaña, 2003; Morgan *et al.*, 2013). As such, services and supports can be better targeted to where they are needed. Thus, the purpose of the study was to explore: What are couples’ perspectives on their needs for health care and support in a subsequent pregnancy after prior early PL and how do these needs change across the pregnancy?

## Materials and methods

### Study setting

**Figure 1.**
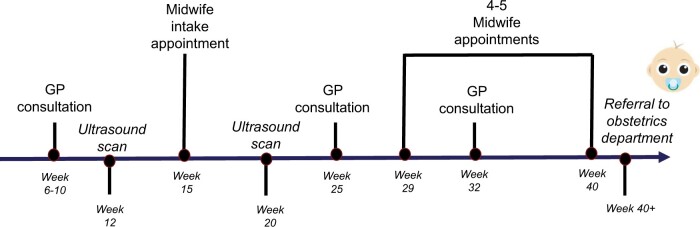
**Recommended standard antenatal care in Denmark.** Includes recommended number of visits with midwife and GP. Actual number of visits may be less than recommended. GP: general practitioner. Adapted from Sundhedsstyrelsen (2022).

In Denmark, in the public healthcare system, women register their pregnancies at their general practitioner’s (GP’s) office. Pregnancy care includes two free ultrasound scans at 12 weeks and 20 weeks and screening for prior or pregnancy-related diseases. Antenatal care is provided by midwives beginning with an intake appointment between week 13 and 15. The majority of antenatal care and contact with a midwife occurs in the second half of pregnancy (20+ weeks) and Fig. 1 shows the standard antenatal care programme in Denmark. Pregnant women are not routinely seen by an obstetrician. Extended antenatal care is available by referral for any pregnant woman in Denmark who meets specific criteria for pregnancy-related complications and/or vulnerability, including previous complicated pregnancy or loss of a child, and includes extra and longer midwife appointments and/or consultations with an obstetrician in the second half of pregnancy. At the time of the study, automatic referral to this programme was not available to patients who had experienced prior PL. Referral to the RPL Unit can be made after three or more consecutive PLs and this includes the ‘Tender Loving Care’ program (Clifford *et al.*, 1997) with ongoing monitoring, scans and support from nurses in the first half of a new pregnancy. For those with the necessary financial resources, scans can be purchased for a fee in private clinics outside the public healthcare system.

### Study design

This was a qualitative, longitudinal dyadic interview study. A longitudinal research design is fitting to explore how needs and experience change across a pregnancy within the same participants (Saldaña, 2003). Dyadic (joint) interviews were conducted with both partners because this method creates a rich, detailed account of an experience as each person can elaborate on and add to what the other has said. This is called the ‘share and compare’ approach where a narrative is created through their interaction and supplementation of each other’s account (Morgan *et al.*, 2013). There is the possibility to express disagreement, which can create an acceptance of differences and mutual resolution (Morgan, 2016). We previously used a dyadic interview method with a sample of couples with RPL and the data were in-depth and nuanced, and feedback about the interview experience from the participants was positive (Koert *et al.*, 2019).

### Data collection

In the current study, four interviews were conducted with each couple: one after a self-reported positive pregnancy test and one in each trimester. The interview guide was developed by the first author and piloted with two couples. The same questions were asked at all four interviews focusing on their experience, coping, and needs. This study reports on the data related to their need for, and access to, health services and support at each stage of the pregnancy (Table 1 shows the relevant interview questions).

**Table 1.** Interview guide examining couples’ needs for and access to health services and support at each phase of pregnancy after prior early pregnancy loss.

Questions asked at each interview:
1. What has been your experience of the X trimester?
2. What healthcare services and supports have you accessed during this time?
3. What, if any, needs have you had for services and supports that haven’t been met?
4. Is there anything else you would like to share?

‘X’ is filled in with positive pregnancy test, or first, second or third trimester as appropriate for the phase of the pregnancy.

Study participants were recruited from The Copenhagen Pregnancy Loss Cohort (COPL, Hartwig *et al.*, 2023), a large prospective cohort study of couples in Denmark initially presenting at acute care in hospital with ultrasound-confirmed PL before gestational age 22 weeks (including ongoing spontaneous PL, missed miscarriage, or anembryonic pregnancy). Women with a molar pregnancy, an ectopic pregnancy or a pregnancy of unknown location were excluded. At the COPL cohort study follow-up meeting ∼6 weeks after the PL, couples were informed of the current study and invited to contact the first author directly if they were interested in being followed during a subsequent pregnancy. No data were shared between the COPL cohort study and current study.

Inclusion criteria included couples with prior PL (1–2 PLs), a self-reported positive pregnancy test and being willing to participate in four joint interviews conducted in English. Informed consent was obtained from each study participant and reviewed at the beginning of each interview.

To ensure trustworthiness of the study findings, the concept of ‘information power’ guided decisions regarding recruitment (Malterud *et al.*, 2016) rather than setting a set number of participants to recruit prior to the study, which is not consistent with the method (Braun and Clarke, 2021). With this concept, study recruitment prioritizes the richness of the data collected over number of participants. If the interviews are in-depth and nuanced with data that is relevant for the research question, they can be assumed to hold more information power. In this study, recruitment and analysis occurred simultaneously so that the co-authors had a sense of the relevance and quality of the data. In total, 15 couples were included in the study with more than sufficient information power after discussion with co-authors of the data collected.

Owing to the coronavirus disease-2019 pandemic, interviews were primarily conducted online by the first author, a trained qualitative interviewer and psychologist. Interviews took place between January 2021 and June 2022. Interviews ranged from 20 to 91 min (mean = 54). The majority of couples participated in all four interviews (Supplementary Table S1). One couple did not complete the fourth interview but provided feedback over email and indicated they wanted their data included in the study.

### Data analysis

Interviews were digitally recorded and transcribed. Data analysis was conducted in two steps: first, a dyadic thematic analysis was used to explore needs in and across couples at each of the four interview time points. Using Braun and Clarke’s (2006) thematic analysis framework, all transcripts from the positive pregnancy test interviews were reviewed several times in order to increase familiarization with the data, sections of transcripts were coded to reflect their meaning using inductive coding (being data driven rather than theory driven) by E.K. E.K. and L.S. met to discuss initial coding and grouping into preliminary data-driven themes. Discussions on the analysis were continued until consensus was reached. These meetings also included a discussion of researcher reflexivity (i.e. the researchers’ positioning and assumptions) in order to increase the rigor of the analysis (Hammarberg *et al.*, 2016). This process was repeated for each of the time points (first, second, third trimesters) (four meetings held in total). Second, using a longitudinal thematic analysis (Saldaña, 2003), codes and themes regarding needs from each of the four time points were reviewed by E.K. and compared with a focus on identifying shifts in needs over time. Two overarching longitudinal themes were developed by E.K. and discussed and agreed upon with all co-authors.

In order to ensure trustworthiness of the findings, analytic steps were informed by the Consolidated Criteria for Reporting Qualitative Research (Tong *et al.*, 2007) and guidelines for qualitative research from Hammarberg *et al.* (2016)’s invited commentary in *Human Reproduction*. This included assembling a multidisciplinary group of co-authors (psychologist, gynaecologists, public health researchers with expertise in infertility, PL, patients’ needs assessment) to bring a wide range of perspectives to the analysis and interpretation of the data. Two meetings were held with all study co-authors to review sections of the interview transcripts and to discuss the coding, themes, and interpretation of the dyadic and longitudinal findings. After the first meeting, in order to reflect discussions and interpretations of the analysis by the team, additional details were included in the dyadic themes from each time point from the original codes to better reflect the nuances of differences in partners. After discussion in the second meeting, the overall thematic map was agreed upon and approved by all co-authors.

### Ethics

Ethics approval was obtained from a University of Copenhagen research ethics committee (approval #504-0252/21-5000). Data protection approval was granted by separate application to the University of Copenhagen (1804669). The study followed the Declaration of Helsinki principles for Medical Research involving Human Subjects. Informed consent was obtained from each participant.

## Results

In total, 15 couples participated in the study. The sociodemographic and reproductive history of the participant couples is outlined in Table 2. Of note is that all prior PLs were early PLs under 12 weeks gestation except for one PL at over 12 weeks. More details about gestational age are provided in Table 2. Two of the couples experienced another early PL during the study. They opted to begin the series of interviews after becoming pregnant again. All other pregnancies followed during the study proceeded to the third trimester.

**Table 2.** Sociodemographic and reproductive history of the participant couples pregnant after prior early pregnancy loss.

Sociodemographics	N (%)
**Age**	Range (median)
Women	28–41 (34)
Partners	27–46 (35)
**Relationship status**	
Married	9 (60)
Common-law	6 (40)
Years together (Range)	2–18

Heterosexual couple	14 (93)
Same sex couple	1 (7)
**Language**	
Both Danish speakers	7 (47)
One Danish speaker	5 (33)
Both non-Danish speakers	3 (20)
**Education** (n = 30)	
High (Masters and above)	13 (43)
Medium (Bachelor or diploma)	15 (50)
Low (High school)	2 (7)

Reproductive history	N (%)

**Pregnancy losses at study start**	
1	6 (40)
2	8 (53)
3	1 (7)
Total	25
**Gestational age at time of pregnancy loss (n = 25)**	
<5 weeks + 6 days	3 (12)
6 weeks + 0 days to 8 weeks + 6 days	13 (52)
9 weeks + 0 days to 11 weeks + 6 days	8 (32)
>12 weeks + 0 days^a^	1 (4)
**Additional pregnancy loss during study**	2 (13)
**Referral to RPL unit during study**	
Total	4 (27)
After 3 PL	3
After 2 PL	1
**Prior children**	
Together	3 (20)
From previous relationship (all men)	3 (20)
**Fertility treatment**	
Yes (for infertility)	4 (27)
Yes (IUI donor sperm)	1 (7)
No	10 (67)

All demographics by couple (n = 15) except where indicated.

PL: pregnancy loss; RPL: recurrent pregnancy loss.

a Gestational age at pregnancy loss was 17 weeks + 0 days.

**Figure 2.**
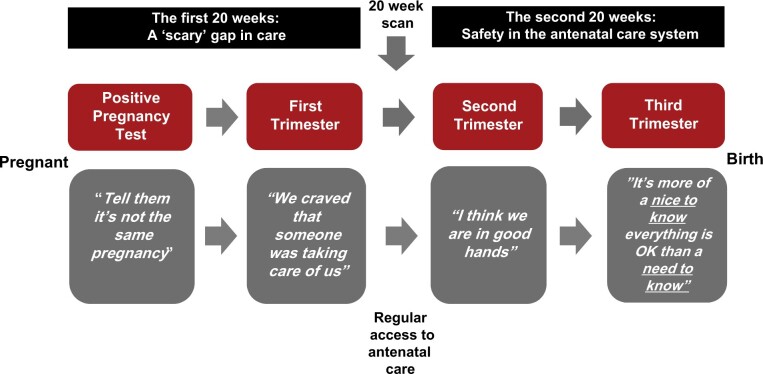
**Thematic map of longitudinal themes and dyadic themes at four time points from pregnancy test to birth.** Black boxes = longitudinal themes. Grey boxes = dyadic themes from each time point. Second trimester interview was after 20-week scan.

Figure 2 outlines a thematic map of the findings. Two longitudinal themes: ‘The first 20 weeks: A ‘scary' gap in care’ and ‘The second 20 weeks: Safety in the antenatal care system’ highlight the shift in expressed needs and access to care after the 20-week scan. Before this point, couples reported experiencing high distress and anxiety about the safety and security of the pregnancy and their unmet needs for care. In the second half the pregnancy, distress was lower and overall needs for care were met given regular access to antenatal care. The specific needs at each of the four time points (e.g. after positive pregnancy test and in each trimester) are represented by a main theme. These themes are described in more detail below. On balance, pregnant women and partners were in agreement about their needs, but differences have been highlighted where present. Direct quotations from participants are in italics and labelled by pregnant woman or partner and couple number. Commonly used phrases are identified with italics and single quotes.

### The first 20 weeks: a ‘scary’ gap in care

#### Positive pregnancy test: ‘Tell them [health care professionals] it’s not the same pregnancy’

The couples described how they did not expect to have a PL in their first pregnancy. Thus, they were able to enjoy the pregnancy with a degree of naiveté, hopefulness, and lack of awareness of the possibility of PL. However, after the PL, they could no longer experience a pregnancy in the same way. In the current pregnancy, both partners experienced anxiety, worry, fear, distress, and uncertainty of having another PL. As a pregnant woman said: ‘*I am scared of everything. I’m scared that something is going to happen*’ (couple 9) and a male partner said: ‘*my mind is full of the same problems (as partner)*’ (couple 8). It was particularly intense in the first 12 weeks of pregnancy. They wanted healthcare professionals to understand that they would never be pregnant in the same way again as that first time, given their history of loss: ‘*Tell them it’s not the same pregnancy*’ (pregnant woman, couple 8). They wanted recognition that because of this history of PL, their treatment should not be the same as if it was a first pregnancy. They wished for healthcare providers to recognize the impact of their history of PL on the current pregnancy and provide sensitive and empathetic care. They wanted any healthcare providers to be aware of their PL(s) and to understand that having a scan, for example, would be very stressful for them, given that in the previous pregnancy they may have discovered the PL at the scan: ‘*If she (person doing scan) said ‘I can see you lost two times’ but maybe here she can just take a little more time like—reassuring us that everything is alright*’ (male partner, couple 8).

If they had any problems, such as bleeding in early pregnancy, they also wanted those providing care to understand there was a valid reason for their anxiety and concern, given their history of PL. One of the partners summarized the essence of this need:

*‘Don’t treat it [current pregnancy] like a first pregnancy. You have to give us some more support because of what we have gone through before. You struggle a lot because you have to push them to give you some support. Otherwise they are like just wait [in case of bleeding] and you’re like no I want to be seen now. You don’t understand the anxiety of someone who has experienced losses’* (male partner, couple 9).

#### First trimester: ‘We craved that someone was taking care of us’

During the first trimester, couples wrestled with feelings of cautious optimism and continued to fear another PL, especially around the gestational week in which they had their previous PL(s). As one participant said, ‘*we live this pregnancy with a bit of distance*’ (pregnant woman, couple 15).

Both partners wished for ongoing monitoring and follow-up that tracked the health of the foetus at this early point in the pregnancy (e.g. scans) and provided the opportunity to share their concerns.

There was considerable variation in access and referrals to care during this period but consistency in the opinion that the available public care did not meet their needs. The couples described an inconsistent system of care where they took scans and monitoring wherever offered before the standard 12-week scan. For example, those that had become pregnant through fertility treatment were offered an early scan in the fertility clinic. Some were offered a scan by a sympathetic gynaecologist they had met in the acute ward during the previous PL. Some were referred for an extra scan by their GP. Others paid for a private comfort scan(s).

*‘It’s not common to get that kind of healthcare [extra scans] after pregnancy losses but it’s because I have been really open about how I’m feeling (to GP)…I am so scared about it getting through with it so it’s because I have talked with (the GP) so that’s how we got that extra help and in the other weeks we pay ourselves for the scans’* (pregnant woman, couple 10). The couples felt unsatisfied by the fragmented nature of care and worried that some issue could be missed because they did not have a consistent healthcare professional following them in early pregnancy. As one partner said, ‘*We craved that someone was taking care of us*’ (male partner, couple 14). They wished for a standardized system for where to call if they had a concern or question rather than spending hours on the phone trying to reach their GP or a nurse or gynaecologist at the acute care ward at the hospital. Partners in particular felt that it would take some of the pressure off of them as the main support person or the one responsible for finding solutions and care (e.g. when pregnant woman was bleeding). Couples wished for individualized care that included sensitivity to their reproductive history of PL, an assessment of their individual needs and an adaptation of approach. They also wished for couple-focused care that acknowledged the impact of PL on both partners. Pregnant women strategized how often the scans were needed and how much uncertainty they could tolerate in between. They wished to have a scan every 1–2 weeks in the first trimester, but this was not available for most. As one pregnant woman said, and others echoed, in the first trimester *‘I try to survive scan to scan’* (pregnant woman, couple 10). They wished for a scan around the gestational week(s) where they had lost the prior pregnancy/pregnancies as they felt particularly worried that they would lose again at this point. Those with two prior PLs thought it was unfair they had to go through another PL before being able to access the ongoing monitoring offered through the RPL programme (3+ PLs).

Given that the relief and reassurance they experienced after a scan was temporary and thus not an overly effective long-term coping strategy, both pregnant women and partners wished for other ways for managing their fears and anxieties between scans. They wanted more information about what to expect and how to cope during the pregnancy:

*‘If you get the information and you know it’s common to have this – it really gives you a lot of comfort. I just think information during all this is key’* (male partner, couple 10).

### The second 20 weeks: Safety in the antenatal care system

#### Second trimester: ‘I think we are in good hands’

There was a shift in the second trimester and second half of the pregnancy after the 20-week scan when couples had more access to antenatal appointments and ongoing monitoring. They felt more ‘safe’ with the pregnancy because they were beyond the week of the pregnancy where they had miscarried in the past and they had access to the ongoing antenatal care they needed. The confirmation of a healthy foetus at the 20-week scan (‘*everything looks normal*’) provided much needed relief and was an important milestone in the pregnancy. As one pregnant woman said, ‘*Even though you have worries you have good days too*’ (pregnant woman, couple 8).

The couples described that there was comfort in starting the antenatal programme (both standard and extended) when there was a standardized schedule of appointments that allowed them to plan the next phase of their pregnancy, leading them to believe ‘*I think we are in good hands*’ (pregnant woman, couple 5). A male partner explained,

*‘The midwife explained everything. You have some worries, she answers those questions. Very clearly explaining and what’s the next step. Yeah you feel like you are really guided you know through the process’* (couple 7).

Participants mentioned they felt relieved they could call the 24-hour nurse/midwife telephone line with any questions or concerns. This telephone line offered by the delivery ward in hospital was available to all pregnant patients in antenatal care, in general accessed after week 20. There was comfort in knowing where to call with a concern or question. Some pregnant women wished for an additional scan, but this was less frequent than in the previous trimester and much easier to request and access. Couples echoed their earlier wish for an individualized approach and sensitivity to their history of PL(s). For example, one couple shared their frustration with an intake appointment with a midwife when they were told the same information again, as if they had not had a prior pregnancy. They had to inform her and this was a painful reminder of the PL.

#### Third trimester: ‘It’s more of a ‘nice to know’ everything is OK than a ‘need to know’

Entering the third trimester provided even more relief for both partners and confidence in the foetus being healthy. Couples described the reassurance that receiving ongoing ‘*proof of life*’ from the baby moving and kicking (i.e. self-monitoring) led them to feel less in need of regular monitoring via the healthcare system. As one partner said of the antenatal care appointments, *‘It’s more of a ‘nice to know’ everything is OK than a ‘need to know’* (male partner, couple 5).

At this point, much of their focus shifted to the upcoming birth rather than the possibility of a loss of the pregnancy. As one partner said, ‘*It’s more of a countdown than a count up now*’ (female partner, couple 1). Whilst partners generally felt they were out of the danger zone, pregnant women highlighted that given their history of PL(s), they would likely not feel truly ‘safe’ until their baby was born: *‘I think it’s true that we would not completely relaxed until he’s there in our arms’* (pregnant woman, couple 5). They wished for their healthcare providers to keep this in mind when providing service.

Although they could likely cope with less monitoring at this point, nevertheless, the couples appreciated the regular monitoring in the standard and extended antenatal care programme, particularly there being a procedure in place if they were having any issues or concerns with the pregnancy. Similarly, whilst they stated their wish to feel it was a ‘*regular pregnancy*’ now, they repeated the importance of receiving individualized care that was sensitive to their earlier PL(s): *‘It feels good that I am in the system like that. All this (prior PL history) is in my file. So when we go in for the birth that they will know all of that’* (pregnant woman, couple 1).

## Discussion

The findings of this longitudinal dyadic interview study provide further support for *The Lancet* series *Miscarriage Matters*’ claim that research on the short- and long-term health consequences of sporadic PL (1–2 PLs) ‘challenge[s] the belief that miscarriage is a single event without wider repercussions’ (The Lancet, 2021a).

The findings showed that couples were ‘*never pregnant in the same way again*’ after PL. Couples described how, in the first 20 weeks, they experienced a ‘*scary*’ gap in care, whilst in the second 20 weeks, they felt safety in the antenatal care system.

This study highlights a gap in care in the weeks of early pregnancy after prior PL before traditional antenatal care begins and before being eligible for referral to the RPL Unit for monitoring (in Denmark 3+ PLs). These are the most distressing weeks of the pregnancy when the couples are uncertain whether they are going to lose the pregnancy again and pregnant women cannot rely on their bodies to confirm the security of the pregnancy as in later weeks when they can feel the baby kicking. Although this study was conducted in Denmark, this gap in early pregnancy care is likely to be similar in other countries (Coomarasamy *et al.*, 2021) and highlights a widespread structural issue. In this study, couples described taking scans and monitoring wherever offered, resulting in a fragmented system of care that did not meet their needs. Instead, they wished for ongoing and consistent monitoring. Thus, when strategizing about a reform of care after PL, consistent with *The Lancet* series, *Miscarriage Matters* (Coomarasamy *et al.*, 2021; The Lancet, 2021a,b), this study suggests that standard PL care should also include early care and monitoring offered in a subsequent pregnancy even after one to two prior PL(s) and supports the need for early pregnancy assessment units (Coomarasamy *et al.*, 2021).

Future research is needed to determine what care in early pregnancy is most effective in meeting needs and reducing distress before making widespread structural changes. For example, the optimal number and frequency of additional scans to reduce anxiety and distress should be examined along with the effectiveness of other forms of monitoring (e.g. access to a medical professional with knowledge of early pregnancy) to provide evidence-based guidelines for early pregnancy care after PL that balances the needs and resources. It may be that one additional standard scan could be offered to all couples with prior PL so they do not need to wait until week 12 to confirm the security of the pregnancy. In the current Danish public health system, there are no standard criteria for referrals for an extra scan for those with one to two prior PL(s), resulting in inconsistent and unequal access to care. Offering a standard early comfort scan for those with prior PL could acknowledge and normalize the need for confirmation and reassurance that the current pregnancy is ‘safe’ and secure, given their history. It would move away from the current system of fragmented care (The Lancet, 2021a). Knowing that they have a scan at a particular week may help couples to plan and manage their distress and anxiety in the time in between because they know there is a check-in point soon.

*The Lancet* series, *Miscarriage Matters*, recommends that after a first miscarriage, women should be offered information on patient support groups, online self-help strategies and information, and screening for mental health issues (The Lancet, 2021b). The current study suggests that *both* women and partners should be offered these support resources into the early period of a new pregnancy. For example, couples wished for more information about what to expect and how to cope during the different pregnancy periods after PL in order to manage their distress. Information-seeking may itself be a coping method in response to anxiety (Charpentier *et al.*, 2022). More research is needed to determine the effectiveness of information provision to decrease anxiety and distress in couples pregnant after PL.

In addition, the findings demonstrate that both partners are negatively impacted and distressed in a pregnancy after PL. Men/partners in this study wished for resources to cope with their own emotional reaction along with supporting their female partner, similar to previous research on men’s experiences after PL (Obst *et al.*, 2020). Thus, there is a need for the development and testing of self-help coping strategies and interventions targeted to both partners so that they can reduce their anxiety and distress and support each other in the early months of pregnancy. Whilst there are interventions to reduce anxiety during pregnancy in pregnant women (e.g. Evans *et al.*, 2020), we do not know if these are effective in pregnant women with a history of PL and specifically in the early pregnancy period when they are most at need. Also, we do not know whether they are relevant for partners and effective in reducing their pregnancy-specific distress. A systematic review of randomized controlled trials of psychological support interventions for pregnant women with a history of PL found no studies that fit the criteria (San Lazaro Campillo *et al.*, 2017), underscoring that this is an under researched area and a gap in knowledge. One coping intervention, the Positive Reappraisal Coping Intervention (Lancastle and Boivin, 2008), is being tested in women with RPL during the waiting period between the positive pregnancy test and the 12-week scan in a new pregnancy (Bailey *et al.*, 2020). In another study, a relaxation intervention was found to reduce pregnancy-related anxiety levels in women with perinatal loss (Duman *et al.*, 2022). There are also interventions to reduce depression, anxiety, or grief after perinatal loss (including PL), but these do not target the subsequent pregnancy period specifically (Jensen *et al.*, 2021; Shaohua and Shorey, 2021).

In the second half of pregnancy, access to antenatal care was higher than the first half of the pregnancy. At this point, pregnant women described that feeling the baby moving gave them ‘*proof of life*’ thus they did not need as much external monitoring (e.g. from scans) because they could monitor the baby themselves. It is not clear whether distress was lower at this point because there was higher access to care or whether higher access to care reduced distress. Likely, this is an interconnected process but couples described the relief in receiving more regular check-ups in the second half of pregnancy and being part of a standardized programme. There is something comforting about knowing there is a standardized programme that can be accessed, as in the RPL’s Tender Loving Care programme (Clifford *et al.*, 1997) of scans and monitoring.

Across the entire pregnancy, couples wished for sensitivity, understanding and empathy from healthcare professionals that a pregnancy after a prior PL is ‘…*not the same pregnancy*’, meaning that their distress and concern about the safety and security of the new pregnancy comes from a valid experience. Previous research shows that interactions with healthcare staff can impact patients’ experience after a PL, underscoring the importance of providing sensitive and supportive care (Galeotti *et al.*, 2022).

Research has shown that women and their partners are dissatisfied with the care they receive after PL and wish for more information, understanding, empathy, and individualized care (Musters *et al.*, 2011, 2013; Meaney *et al.*, 2017; van den Berg *et al.*, 2018; Koert *et al.*, 2019). The present study highlights that these wishes extend into a new pregnancy. For example, couples wanted their concerns acknowledged and the care they received adapted to their history of PL. Healthcare professionals could normalize couples’ concerns in early pregnancy whilst being cautious not to dismiss them and provide hope that the second half of pregnancy can be less stressful. Additional training for healthcare professionals is needed to create more awareness that a subsequent pregnancy after PL is not without concern. For these couples, it is not as simple as ‘just try again’ for another pregnancy (The Lancet, 2021a).

### Strengths and limitations

To the best of our understanding, this is the first qualitative longitudinal study to follow couples through a new pregnancy after PL. This method is appropriate in order to understand the nuances of shifts across the pregnancy. This study is one of few on this topic that includes both partners in the dyadic interview research design. PL impacts both partners; thus, it is fitting that both of their perspectives were included.

The participants were self-selected. Although the majority participated in every interview, one couple chose not to complete the final interview and provided feedback via email that they wished to have their data included in the study. For three couples, interviews were combined owing to timing and availability (e.g. positive pregnancy test and first trimester; Supplementary Table S1).

Couples were interviewed together and each partner was asked about their opinion and experience. Mutual influence might have been a factor although there were instances where partners disagreed with each other. The dyadic interview method sees interviewing couples together as an advantage to create more complete and rich data, where the participants supplement each other by adding details and reminding each other of experiences (Morgan, 2016). Creating a shared narrative about a shared experience also can provide some benefit to the participants, bringing them together and uniting them in the experience or mutual resolution of difference (Morris, 2001; Morgan, 2016). All of the participants said that it had been helpful to have these discussions with their partner in the interviews. However, we acknowledge that there might have been perspectives that could not be shared and/or that this method precluded some participants from participating (e.g. single women or those where partners disagreed about participating in the study).

Couples were interviewed in English. This might have prevented some Danish couples from participating although many Danish people know and speak English well. The benefit may be that there was heterogeneity in the sample (e.g. less than half of couples participating were both Danish, one-third had one non-Danish partner) potentially presenting a wide range of experiences. In order to manage the methodological challenges in cross-language qualitative interviews, the interview questions were developed by bilingual co-authors and pilot tested with the first two couples (Squires, 2009). These pilot interviews were included in the analysis given they generated rich data. In addition, this was a well-educated sample with only 7% having a low education level, perhaps a result of the English language requirement.

This is a qualitative study; thus, the findings cannot be generalized to all couples pregnant after PL. However, there is increasing recognition of the value of qualitative research in health care and services research, where in-depth interviews can provide important insights into a patient’s experience with, and access and barriers to care (Mays and Pope, 2020). Although this study was conducted in Denmark, the findings highlight unmet needs in a public healthcare system model where antenatal care provides only one standard scan at 12 weeks and RPL Unit early pregnancy monitoring is only offered to those with three or more PLs. This points to the need for early pregnancy assessment units and the possible benefit of having an organized, multidisciplinary skilled team caring for early pregnancy complications (Coomarasamy *et al.*, 2021).

## Conclusion

The findings underscore that an early PL creates increased need for monitoring and care in a subsequent pregnancy. This study highlights a gap in pregnancy care for those with a history of one or two PL(s), given that their need for monitoring and support is high in the early weeks of a new pregnancy before they have access to antenatal care, and before they have had multiple PLs and can be referred to the RPL unit. This gap in early pregnancy care highlights a widespread structural issue that is likely present in many other countries (Coomarasamy *et al.*, 2021). Future research should examine what is the most effective form of care and monitoring to reduce distress in the early pregnancy period and if improved care would be cost-beneficial. The findings suggest that additional efforts are needed to increase healthcare professionals’ awareness and sensitivity to couples’ experience and needs during a pregnancy after PL, and to develop and test self-help interventions that include strategies to help both partners manage their distress and anxiety, and support each other in early pregnancy after PL.

## Supplementary Material

hoad032_Supplementary_Data

## Data Availability

The data underlying this article cannot be shared publicly due to protecting the privacy of individuals who participated in the study under the agreement that their data would be anonymous and the transcripts would not be shared, consistent with the ethical requirements for collection of interview data at the University of Copenhagen. The audio-recordings were destroyed at study completion in keeping with the requirements for the General Data Protection Regulation agreement.
