# Supplementary material for: ‘You’re never pregnant in the same way again’: prior early pregnancy loss influences need for health care and support in subsequent pregnancy
Source: Hum Reprod Open. 2023 Aug 1;2023(3):hoad032. doi: 10.1093/hropen/hoad032 (PMC10412407; doi:10.1093/hropen/hoad032)
Supplement: hoad032_Supplementary_Data [file hoad032_supplementary_data.docx]

**Supplementary Table S1** Interview participation by couple.

| **Couple number** | **Positive pregnancy test** | **End of**  **first trimester**  **After 12 week scan** | **Second trimester**  **After 20 week scan** | **Third trimester**  **After 29 weeks** |
| --- | --- | --- | --- | --- |
| 1 | X | X | X | X |
| 2 | X | X | X | X |
| 3 | X | X | X | X |
| 4 |  | X | X | X |
| 5 | X | X | X | X |
| 6 | X | X | X | X |
| 7 | X | X | X | X |
| 8 | X | X | X | X |
| 9 | X | X | X | X |
| 10 | X | X | X | X |
| 11 | X | X | X | X |
| 12 | X |  | X | X |
| 13 |  |  | X |  |
| 14 | X | X | X | X |
| 15 | X | X | X | X |
| TOTAL  15 | 13 | 13 | 15 | 14 |

Note: Blank squares indicate missing interviews. Couple 13 chose not to complete the final interview and provided feedback via email that they wished to have their data included in the study. For a few other couples, a missing interview was combined with the subsequent interview due to timing and availability.
